# Reporting quality of the 2014 Ebola outbreak in Africa: A systematic analysis

**DOI:** 10.1371/journal.pone.0218170

**Published:** 2019-06-25

**Authors:** Nina Huynh, Andrea Baumann, Mark Loeb

**Affiliations:** 1 Global Health Office, McMaster University, Hamilton, Ontario, Canada; 2 Department of Pathology and Molecular Medicine, McMaster University, Hamilton, Ontario, Canada; 3 Department of Health Research Methods, Evidence, and Impact, McMaster University, Hamilton, Ontario, Canada; 4 Michael G DeGroote Institute for Infectious Disease Research, McMaster University, Hamilton, Ontario, Canada; Tulane University, UNITED STATES

## Abstract

The objective of this study was to conduct a systematic analysis of the reporting quality of the Ebola Virus Disease (EVD) outbreak in West Africa from 2014–2018 using the Modified STROBE statement. We included studies on the 2014 EVD outbreak alone, limited to those on human patients in Africa. We searched the following databases (MEDLINE, EMBASE, and Web of Science) for outbreak reports published between 2014–2018. We assessed factors potentially associated with the quality of reporting. A total of 69 of 131 (53%) articles within the full-text review fulfilled our eligibility criteria and underwent the Modified STROBE assessment for analyzing the quality of reporting. The Modified STROBE scores of the included studies ranged from 11–26 points and the mean was found to be 19.54 out of 30 with a standard deviation (SD) of ± 4.30. The top three reported Modified STROBE components were descriptive characteristics of study participants, scientific background and evidence rational, and clinical significance of observations. More than 75% of the studies met a majority of the criteria in the Modified STROBE assessment tool. Information that was commonly missing included addressing potential source of bias, sensitivity analysis, further results/analysis such as risk estimates and odds ratios, presence of a flowchart, and addressing missing data. In multivariable analysis, peer-reviewed publication was the only predictor that remained significantly associated with a higher Modified STROBE score. In conclusion, the large range of Modified STROBE scores observed indicates variability in the quality of outbreak reports for EVD. The review identified strong reporting in some areas, whereas other areas are in need of improvement, in particular providing an important description of the outbreak setting and identifying any external elements (potential biases and confounding factors) that could hinder the credibility of the findings.

## Introduction

Since 1976, Ebola Virus Disease (EVD) has persisted as a rare and deadly illness that has caused socioeconomic disruptions worldwide due to a fatality rate ranging from 25% to 90% in previous outbreaks[1]. Notably, the 2014–2015 epidemic in Africa severely impacted Guinea, Sierra Leone and Liberia and was 11 times larger than all of the past outbreaks combined [2]. Numerous studies have demonstrated that many affected countries were ill-equipped to handle the magnitude of the 2014 epidemic because they lacked the clinical capacity and resources; inadequate funds were invested into the public health system; and surveillance systems were poorly governed[3–5].

Outbreaks like these are commonly reported as descriptive observational studies which include case reports, surveillance, and cross-sectional studies to evaluate infection control interventions[6]. High-quality informative reports are interpreted as containing all of the necessary documentation about the relevant study (e.g. outbreak location, pathogen type, number of individuals exposed and infected). This information can act as the fundamental source of epidemiological data for assessing the health of populations; determining how outbreaks can be managed; and improving prevention measures of communicable diseases[7,8].

A movement towards better reporting standards began in the 1990’s with the development of evidence-based medicine due to the recognition that inadequate reporting potentially leads to ineffective healthcare policies and/or treatments, putting patients at risk of adverse effects[9]. Since then, guidelines have been written for many different types of studies to increase the clarity in reporting and credibility of published literature[10]. Examples of standardized guidelines include: CONSORT (Consolidated Standards of Reporting Trials)[11], QUOROM (for meta-analyses of randomized trials)[12], STROBE (Strengthening the Reporting of Observational Studies in Epidemiology)[13, 14], and REMARK (Reporting Recommendations for Tumour Marker Prognostic Studies)[15]. Despite the progress made toward higher-quality reporting, recent literature demonstrates that major methodological weaknesses still exist[16–26].

On a global scale, scientists have assessed the reporting quality of outbreaks without the use of a standardized guideline where quality has been graded based on the consistency and completeness of data collected. Examples of this include the study on compiling the world’s first worldwide database on nosocomial outbreak[27] and the evaluation of Foot-and-Mouth Disease outbreaks in mainland South-East Asia from 2000 to 2010[28]. This led to a review where the newly developed Modified STROBE statement approach was used to systematically assess the quality of Influenza outbreak reports[29]. Compared to the original STROBE criteria, a 22-item checklist[13,14], the Modified STROBE assessment tool has additional criteria that includes outbreak characterization, location, and organization of patient data.

Currently, no studies have evaluated the caliber of reporting outbreaks for EVD, demonstrating the novelty of this review[30–33]. The objective of this study was to conduct a systematic analysis of the reporting quality of the EVD outbreak in West Africa from 2014–2018 using the Modified STROBE statement.

## Methods

### Eligibility criteria

We included studies on the 2014 EVD outbreak alone, limited to those on human patients in Africa. In addition, eligible reports needed to describe one or more of the following: onset of the outbreak; clinical manifestations; and control measures of specific diagnostic testing. Although outbreak reports that met our inclusion criteria could have conducted transmission modelling, we excluded studies that were not outbreak reports but only transmission models. Randomized trials and intervention studies were also excluded from the review because they did not include components of outbreak reports.

### Search strategy and data collection

**Fig 1:**
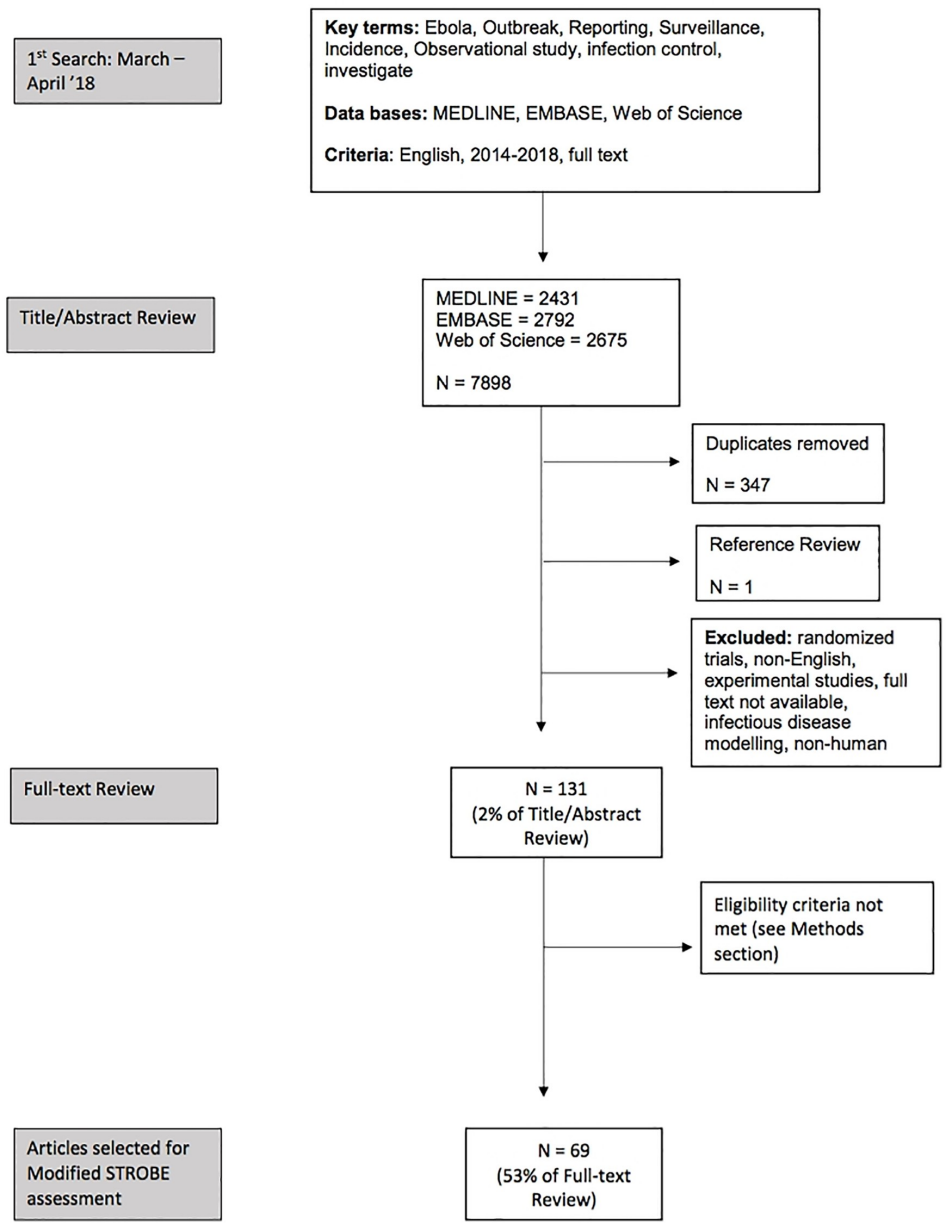
Flow diagram of search strategy and outbreaks included based on eligibility criteria.

We searched the following databases (MEDLINE, EMBASE, and Web of Science) for outbreak reports published between 2014–2018 using the combination of search terms seen in the Table in S1 Table. The search strategy used is described in Fig 1.

### Quality assessment

The Modified STROBE (a 30-item assessment tool that relates to the title/abstract, introduction, methods, results, and discussion of articles) was used to effectively analyze key factors of outbreak reporting[32]. The methods section is further divided into components that correspond to outbreak characteristics; outbreak setting; and organization of patient data to systematically analyze the quality of reporting. Data was extracted from each outbreak report based on the criteria within the Modified STROBE statement, where each individual component is given a worth of one point. A total score (i.e. completeness of reporting) out of 30 points was assigned for each outbreak report as indicated in the S2 Table. The 69 articles included in the review were analyzed by one researcher who has experience using critical appraisal techniques.

To analyze the proportion of articles that accurately met key component of the Modified STROBE checklist, a post-hoc assessment was performed. We identified the following items to be fundamental in an outbreak report: present key elements of study design early in report (3A); case definitions for outbreaks were included (3G); provide eligibility criteria for selection of cases, participants and/or controls (3J); describe any efforts to address potential sources of bias (3L); and give characteristics of study participants (4B).

### Predictor variables

To assess factors potentially associated with the quality of reporting (i.e. Modified STROBE score), the following predictor variables were selected for assessment prior to the study: publication year, author affiliation (academic institution vs. non-academic [e.g. public health agencies, non-governmental organizations]), publication type (peer-reviewed vs. epidemiological report), and outbreak setting (hospital vs. community). We treated publication year as a binary variable, that is, either prior to after the year 2015, due to the substantial amount of outbreak reports published following the outbreak in Africa. Furthermore, we hypothesized that authors obtaining support from public health officials led to higher quality reports. Author affiliation was determined by the first author. In the case that the outbreak report involved authors from public health and academic institutions, the second author determined the author affiliation. Similarly, we predicted that higher quality reports were from peer-reviewed articles and we sought to observe if this is true. On a similar note, we projected that outbreaks that took place at hospitals were reported more accurately compared to those in the community.

### Statistical analysis

We used descriptive statistics to summarize the results of the Modified STROBE scores. For the univariate analysis, a two-tailed t-test was conducted to determine significant predictor variables with p-value <0.05. In terms of the multivariable analysis, a backward stepwise linear regression model was applied to analyze predictor variables associated with better reporting quality. This was done by eliminating non-significant covariates one by one using 5% significance until a final model was obtained. All statistical analysis was conducted using IBM SPSS Statistics Version 25 (SPSS Inc., Armonk, NY, USA).

### Reliability and validity tests

A Cronbach’s alpha test was conducted to measure reliability or internal consistency for the Modified STROBE assessment tool. To measure construct validity, a convergent correlation test was carried out. All the included articles (n = 69) were first critically assessed using the ORION (Outbreak Reports and Intervention studies of Nosocomial infection) statement[6], a 22-item checklist that has a similar outbreak investigation guideline to the Modified STROBE tool. The empirical relationship between the Modified STROBE scores and the scores obtained from using the ORION statement was compared through a bivariate Pearson analysis.

## Results

### Quality assessment

A total of 69 of 131 (53%) articles within the full-text review fulfilled our eligibility criteria and underwent the Modified STROBE assessment for analyzing the quality of reporting [34–102].

The Modified STROBE scores of the included studies ranged from 11–26 points and the mean was found to be 19.54 out of 30 with a standard deviation (SD) of ± 4.30. The distribution of scores is shown in S1 Fig. As indicated in Table 1, all reports provided quantitative data on infected individuals (2C) such as the reported number of suspected and confirmed patients. In terms of frequently reported Modified STROBE components, the top three items commonly reported were: descriptive characteristics of study participants (4B), scientific background and evidence rational (2A), and clinical significance of observations (5A). More than 75% of the studies met majority of the criteria in the Modified STROBE assessment tool. However, information that was commonly missing included: addressing potential source of bias (3L), sensitivity analysis (3O), further results/analysis such as risk estimates and odds ratios (4E), presence of a flowchart (4A), and addressing missing data (3N)(Table 1).

**Table 1:** Components of the Modified STROBE checklist and proportion of articles (n = 69) accurately reporting each item [13].

Modified STROBE Item	Component Description	n(%) Accurately Reported
**1) Title and Abstract**	A) Either title, abstract or both sections clearly indicated study design	60(87)
	B) Study’s focus and investigation details within title, abstract or both sections (e.g. Ebola subtype, geographic location, setting) were clearly elicited	64(93)
	C) Informative summary provided in the abstract discussing steps taken along with investigation findings	52(75)
**2) Introduction**	A) Scientific background, evidence, rationale provided for reporting and conducting investigation	67(97)
	B) Specific objectives for study stated, included pre-established hypothesis if applicable	63(91)
	C) Specific quantiles provided: for example, number of outbreaks/communities reported, number of patients from Ebola outbreak (suspected, confirmed, total)	69(100)
	D) A timeline of the study was provided: includes start/finish dates of conducted investigation or outbreak	57(83)
**3) Methods**	A) Present key elements of study design early in report	59(86)
	B) Was decision to report promoted by any outcome data?	63(91)
**Outbreak Characteristics**	C) Number of patients admitted during outbreak	59(86)
	D) Distribution provided for patient demographics	48(70)
	E) Proportion admitted from other hospitals, wards, communities	32(46)
	F) Potential risk factors for acquiring organism included	47(68)
	G) Case definitions for outbreaks were included	48(70)
	H) Proportions of patient outcomes were included (e.g. ICU, hospitalization, mortality)	58(84)
**Outbreak location/setting**	I) Description of unit, hospital, community	28(41)
**Organization of patient and sample data**	J) Provide eligibility criteria for selection of cases, participants and/or controls (more for cohort/case control)	51(74)
	K) Provide number of exposed/unexposed (cohort) or controls per case (case-control)	42(61)
	L) Describe any efforts to address potential sources of bias	2(3)
	M) Explain how the final study size was arrived at (for patient/case count)	30(43)
	N) Explain how missing data were addressed	17(25)
	O) Describe any sensitivity analysis	6(9)
**4) Results**	A) Consider use of a flow diagram to depict patient or participant count at each stage of investigation	13(19)
	B) Descriptive characteristics of study participants (e.g. demographics, clinical, social) information on exposures and any other associative factors	68(99)
	C) Timeline: chart to display duration of patient stay, date of detecting organisms	59(86)
	D) Consideration of any confounding variables (e.g. use of antibiotics, length of stay changes)	15(22)
	E) Further results and analysis: if applicable, provide unadjusted and confounder-adjusted estimates with confidence intervals(e.g. risk estimates, odds ratios)	16(23)
**5) Discussion**	A) Clinical signification of observation was considered and hypotheses were reviewed in relation to the findings	66(96)
	B) Discuss limitations of study, accounting for any potential bias	38(55)
	C) Discussed generalizability (external validity) of findings and applicability with current evidence)	52(75)

In regard to the post-hoc assessment, majority of papers satisfied four of the five key components within the Modified STROBE checklist. The only criteria that was commonly missed was describe any efforts to address potential sources of bias (3L), as only two reports sufficiently mentioned this information (Table 2).

**Table 2:** Key components of the Modified STROBE checklist and proportion of articles (n = 69) accurately reporting each item.

Modified STROBE Item	Component Description	n(%) Accurately Reported
3A	Present key elements of study design early in report	59(86)
3G	Case definitions for outbreaks were included	48(70)
3J	Provide eligibility criteria for selection of cases, participants and/or controls (more for cohort/case-control)	51(74)
3L	Describe any efforts to address potential sources of bias	2(3)
4B	Give characteristics of study participants (e.g., demographic, clinical, social) + information on exposures and any other associative factors	68(99)

### Factors associated with high-quality reporting

Three variables (publication year, journal type, and author affiliation), were found to be significantly associated with a higher Modified STROBE score in the univariate analysis (Table 3).

**Table 3:** Univariate and multivariate analysis of predictors for reporting quality.

Predictor Variables	Comparison Groups	n (%)	Modified STROBE Mean Score (SD)	Mean Difference (95% CI)	P-values (Univariate Analysis)	P- values (Multivariate regression model)
Publication Year	>2015	25(36)	21.84(3.03)	3.61(1.81 to 5.41)	<0.001	0.089
	≤2015	44(64)	18.22(4.40)			
Journal Type	Peer-reviewed	46(67)	21.48(3.29)	5.83(4.13 to 7.52)	<0.001	0.001
	Epidemiologic Report	23(33)	15.65(3.41)			
Outbreak Setting	Hospital	47(68)	20.02(4.63)	1.52(-0.69 to 3.73)	0.174	0.812
	Community	22(32)	18.50(3.41)			
Author Affiliation	Academic Institution	23(33)	21.39(3.50)	2.78(0.83 to 4.74)	0.01	0.943
	Non-academic Institution	46(67)	18.61(4.41)			

Cl (confidence intervals); SD (standard deviation)

Of the 69 reports included, 25(35%) were published from 2016 to 2018 (i.e. >2015) (S2 Table). These had significantly higher Modified STROBE scores compared to reports published prior to 2015(i.e. ≤2015) (MD 3.61, 95% Cl 1.81–5.41, P = <0.001). We found that 46 (67%) of the studies that were peer-reviewed publications had significantly higher scores in comparison to non-peer reviewed public health epidemiological reports (MD 5.83, 95% Cl 4.13–7.52, P = <0.001). Similarly, 23(33%) of the included outbreak reports did not have any affiliation with public health agencies. We observed significantly higher scores from reports published through academic institutions, in contrast to public health agencies (MD 2.78, Cl 95% 0.83–4.74, P = 0.01). The final predictor, outbreak setting (hospital vs. community) was not found to be a significant predictor for higher Modified STROBE score (P = 0.174).

In the multivariable analysis, peer-reviewed publication was the only predictor that remained significantly associated with a higher Modified STROBE score (P = 0.001).

### Reliability and validity tests

The Cronbach’s alpha test was calculated to be 0.771 and the correlation coefficient value from the convergent validity analysis was found to be 0.832. This demonstrates a strong positive correlation value, indicating the critical appraisal tool measures what it is intended to and has high construct validity.

## Discussion

The main finding from this study was that of the 69 articles assessed, the reports on average met only a modest number of criteria (66%) within the Modified STROBE assessment tool. The total Modified score out of 30 points ranged from 11 to 26. We also found in the multivariable analysis that peer-reviewed articles were associated with a significantly higher Modified STROBE score in comparison to epidemiological reports. To assist in the interpretation of this analysis, it is fundamental to note that we analyzed the completeness of reporting through the total Modified STROBE score and not methodological quality. Hence, items were recorded based on sufficient information to conduct appraisal.

Certain items on the Modified STROBE assessment tool such as sensitivity analysis(3O), may not be necessary for some studies, based on their objective which explains the fact that only a moderate number were met. Items within the Modified STROBE that are considered crucial for reports include outbreak characteristics and description of outbreak location as the inclusion of this information will assist in future outbreak management. The criteria that was commonly missed within the key Modified STROBE components was the identification to address potential sources of bias (3L).

It is not surprising that peer-reviewed articles were found to be associated with higher Modified STROBE scores. A 2015 survey done by publishing research consortium demonstrated that 82% of researchers agreed that the peer-review process is pivotal to the control of scientific communication and improving the quality of published literature[103]. Thus, journal requirements play a fundamental role in the dissemination of research. The peer-review process and the use of reporting guideline requirements are expected to improve the quality of research. Hence, it is highly recommended authors submit papers to journals who have a peer-review process in place in order to improve the quality of manuscripts.

Two predictor variables (publication date and author affiliation) were found to have superior Modified STROBE scores when assessed respectively as an independent predictor but not in the multivariate regression model. The most obvious explanation for publication date is that reports published after 2015 had the time and data advantage. In addition, it is important to note that 23 out of 25(92%) of the articles post 2015 were peer-reviewed and did not contain any public health affiliation. This may have acted as a confounding factor and was accounted for in our multivariate analysis. In regard to author affiliation, there could be multiple factors that influence the reason why academic institutions was found to be associated with a higher Modified STROBE score, for instance funding availability, abiding by institutional regulations, and academic capacity and training.

This is the second time the Modified STROBE assessment tool has been used to evaluate the reporting quality of outbreaks; Lo et al[29] was the first to utilize the appraisal tool for influenza outbreaks. Similar to our findings, Lo et al[29] stated that very few reports provided crucial information on patient characteristics and addressing limitations that could potentially bias the findings. As well, our mean Modified STROBE score (19.54) was similar to their study[29], indicating a new potential trend of excluding fundamental outbreak characteristics may be seen in similar pathogen outbreaks. This not only suggests the generalizability of our modified assessment tool towards other pathogens and outbreak settings, it also reiterated the demand for explicit reporting guidelines for outbreak reports.

One strength of this study is that the reliability and validity test indicated that the Modified STROBE assessment tool was an appropriate instrument to measure the quality of reporting outbreaks. The Cronbach’s alpha test was found to be 0.771, which is within the acceptable range of 0.70–0.90 as demonstrated by various studies[104–106]. This indicates that all the items within the critical appraisal tool are interrelated and measure the same construct. The convergent validity test showed a high positive correlation between the two appraisal tools, ORION and Modified STROBE (correlation coefficient of 0.832), which indicates high construct validity and strengthens the application of this instrument for future use[107,108]. Other strengths of this review include an extensive search strategy and a methodology approach based on the STROBE statement, which has been published in over 122 journals[13,14]. The International Committee of Medical Journal editors have endorsed the STROBE statement as a universal requirement for manuscripts submitted to biomedical journals[13,14]. In addition, conducting a backward stepwise regression model reduces the risk of multicollinearity and overfitting, which are frequently seen in this type of analysis[109–111].

We acknowledge that one limitation of our paper was that we did not include an assessment of the gray literature. This remains an important gap in the academic analysis of outbreak management. We would encourage investigators of outbreaks to develop standardized data fields and additional resources to support data collection that could would facilitate bringing investigations to publications. The Health Internetwork Access to Research Initiative is an example of an initiative developed by the WHO and biomedical healthcare journals to allow complementary or low priced online access to key biomedical journals for developing countries[112–114]. Equally as important to increasing access to health research is containing an efficient number of trained personnel to document and facilitate the post hoc analysis. Thus, we recommend once an outbreak has been reported, mobilized teams should contain trained personnel to assist in data collection. Public health agencies should also be encouraged to publish their results as an expectation of their role in data sharing. In addition, restricting studies to the use of only English text, and narrowing the scope to countries in only one continent (Africa), may not be an accurate comprehensive representation of outbreaks reports. On a similar note, it was difficult to distinguish mutual exclusivity between comparison groups for author affiliation based on first corresponding author. In the review, 46 (67%) of the included articles had affiliations with both academic institutions and public health organizations. It is also important to emphasize that the resources available for reporting outbreaks have not been considered in this analysis.

Outbreaks are a complex situation and multiple external environmental factors–resource availability, public and political climate, response coordination, and development of a skilled workforce—not only directly impact the quality of research collected, but contributes to the spread of the EVD epidemic [3–5]. This clarifies the difficulty in executing routine laboratory analytics and the substantial number of reports with missing data. Hence, this is why it is important to prioritize data collection during an outbreak response. It is clear that there were great challenges in investigating EVD outbreaks and in such circumstances it can be impossible to meet reporting standards. The purpose of this paper is not to disparage those investigators but to draw attention to the need for adequate resources both for outbreak investigation and for reporting.

In summary, the large range of Modified STROBE scores observed indicates the variability in the quality of outbreak reports for EVD. The review identified strong reporting in some areas, whereas other areas are in need of improvement, in particular providing an important description of the outbreak setting and identifying any external elements (potential biases and confounding factors) that could hinder the credibility of the findings. This review acts as a call of action for international organizations (global public health corporations, academic institutions, national non-government agencies) to extend support towards standardizing outbreak reporting, prioritizing data collection, and increasing field epidemiology training programs in developing countries. The Centre for Disease Control’s Field Epidemiology program has shown to be an effective approach towards training residents in developing countries to analyze, collect, and interpret disease information [115]. The adaption of the Modified STROBE checklist to this program could enhance sound infection control policies, leading to better reporting outcomes in the future. Several systematic reviews have documented the positive impacts reporting guidelines have had on quality of reporting, demonstrating the potential effects of the Modified STROBE checklist [116–120]. Therefore, better adherence to the Modified STROBE would increase clarity to research findings, facilitate evidence-informed planning towards future outbreak management, and ultimately aid in the synthesis of policy and practice.

## Supporting information

S1 Table Modified STROBE scores for individual outbreak reports (n = 69)^34-102^. (DOCX)

S2 Table Key Word search strategy in MEDLINE/EMBASE/Web of Science in April 2018†. (DOCX)

S1 Fig Distribution of Modified STROBE score for the 69 outbreak reports. (TIF)

S1 File Strobe data. (XLSX)
