# Supplementary material for: Reporting quality of the 2014 Ebola outbreak in Africa: A systematic analysis
Source: PLoS One. 2019 Jun 25;14(6):e0218170. doi: 10.1371/journal.pone.0218170 (PMC6592536; doi:10.1371/journal.pone.0218170)
Supplement: S1 Table — (DOCX) [file pone.0218170.s001.docx]

**S2 Table.**  **Key Word Search Strategy in MEDLINE/EMBASE/Web of Science in April 2018. †**

| **#** | **Key Word Searches** |
| --- | --- |
| 1 | Ebola* |
| 2 | Outbreak* |
| 3 | Reporting* |
| 4 | Surveillance* |
| 5 | Incidence* |
| 6 | Observational Study* |
| 7 | Infection Control* |
| 8 | Investigate* |
| 9 | #2 or #3 or #4 or #5 or #6 or #7 or #8 |
| 10 | #1 AND #9 |

†As mentioned in the Methods Section and in Figure 1, search terms were used in combination of “OR” and “AND” prior to eliminating duplicates, non English language, non full-text articles. Articles then underwent Title and Abstract review before being screened based on our eligibility criterion (eg, non-intervention studies, human patients only, location and within the 2014-2018 time range). A full-text review was conducted for the remaining articles before undergoing the Modified STROBE assessment tool.
