# Supplementary material for: Reporting quality of the 2014 Ebola outbreak in Africa: A systematic analysis
Source: PLoS One. 2019 Jun 25;14(6):e0218170. doi: 10.1371/journal.pone.0218170 (PMC6592536; doi:10.1371/journal.pone.0218170)
Supplement: S2 Table — (DOCX) [file pone.0218170.s002.docx]

**S1 Table.** **Modified STROBE scores for individual outbreak reports (n = 69).^34-103^**

| **Name of Study** | **Author, Year, Publishing Journal** | **Modified STROBE score (/30)** |
| --- | --- | --- |
| Analysis of patient data from laboratories during the Ebola virus disease outbreak in Liberia, April 2014 to March 2015^34^ | Y Furuse et al. 2017, PLOS Neglected Tropical Diseases | 19 |
| The Transmission Chain Analysis of 2014-2015 Ebola Virus Disease Outbreak in Koinadugu District, Sierra Leone: An Observational Study^35^ | IS Muoghalu et al. 2017, Frontiers in Public Health | 23 |
| Clinical Features of and Risk Factors for Fatal Ebola Virus Disease, Moyamba District, Sierra Leone,  December 2014–February 2015^36^ | Y Haaskjold et al.2016, Emerging Infectious Disease | 20 |
| Clinical Illness and Outcomes in Patients with Ebola in Sierra Leone^37^ | JS Schieffelin et al. 2014, NEJM | 21 |
| Clinical presentation, biochemical, and haematological parameters and their association with outcome in patients with Ebola virus disease: an observational cohort study^38^ | L Hunt et al. 2015, Lancet | 23 |
| Clinical Presentation of Patients with Ebola Virus Disease in Conakry, Guinea^39^ | El Bah et al. 2015, NEJM | 26 |
| Control of Ebola Virus Disease — Firestone District, Liberia, 2014^40^ | EJ Reaves et al. 2014, MMWR | 16 |
| Controlling the Last Known Cluster of Ebola Virus Disease — Liberia, January–February 2015^41^ | T Nyenswah et al. 2015, MMWR | 14 |
| Decreased Ebola Transmission after Rapid Response to Outbreaks in Remote Areas, Liberia, 2014^42^ | KA Lindblade et al. 2015, Emerging Infectious Diseases | 21 |
| Distinguishing epidemiological features of the 2013–2016 West Africa Ebola virus disease outbreak^43^ | JM Schultz et al. 2016, Disaster Health | 15 |
| Ebola Epidemic — Liberia, March–October 2014^44^ | T Nyenswah et al. 2014, MMWR | 21 |
| Ebola Transmission Linked to a Single Traditional Funeral Ceremony —Kissidougou, Guinea, December, 2014–January 2015 ^45^ | KR Vicotry et al. 2015, MMWR | 17 |
| Ebola Viral Disease Outbreak — West Africa, 2014^46^ | MG Dixon et al. 2014, MMWR | 13 |
| Ebola Virus Disease among Children in West Africa^47^ | WHO Ebola Response Team 2015, NEJM | 14 |
| Update: Ebola Virus Disease Epidemic — West Africa, December 2014^48^ | CDC 2014, MMWR | 14 |
| Ebola Virus Disease in Health Care Workers — Guinea, 2014^49^ | M Gruinell et al. 2014, MMWR | 23 |
| Ebola Virus Disease in the Democratic  Republic of Congo^50^ | G Magana et al. 2014, NEJM | 20 |
| Ebola Virus Disease in West Africa — The First 9 Months of the Epidemic and Forward Projections^51^ | WHO Ebola Response Team 2014, NEJM | 26 |
| Ebola Virus Disease Outbreak — Nigeria, July–September 2014^52^ | F Shuaib et al. 2014, MMWR | 14 |
| Update: Ebola Virus Disease Outbreak — West Africa, October 2014^53^ | CDC 2014, MMWR | 11 |
| Ebola Virus Disease — Sierra Leone and Guinea, August 2015^54^ | S Hersey et al. 2015, MMWR | 17 |
| Ebola Virus Epidemiology and Evolution in Nigeria^55^ | OA Folarin et al. 2016, Infectious Diseases Society of America | 20 |
| Ebola Virus Outbreak Investigation, Sierra Leone, September 28–November 11, 2014^56^ | H Lu et al. 2015, Emerging Infectious Diseases | 26 |
| Epidemiological and Surveillance Response to Ebola Virus Disease Outbreak in Lofa County, Liberia (March-September, 2014); Lessons Learned^57^ | KI Koudaio et al. 2015, PLOS | 20 |
| Epidemiological features and trends of Ebola virus disease in West Africa^58^ | L Wang et al. 2015, International Journal of Infectious Diseases | 13 |
| Epidemiological profile of the Ebola virus disease outbreak in Nigeria, July-September 2014^59^ | EO Musa et al. 2015, Pan African Medical Journal | 22 |
| Ebola Virus Disease Outbreak — West Africa, September 2014^60^ | CDC 2014, MMWR | 13 |
| Epidemiology of Epidemic Ebola Virus Disease in Conakry and Surrounding Prefectures, Guinea, 2014–2015 ^61^ | A Rico et al. 2016, Emerging Infectious Diseases | 21 |
| Epidemiology of Ebola Virus Disease in the Western Area Region of Sierra Leone, 2014-2015^62^ | L Lamanu et al. 2017, Frontiers in Public Health | 24 |
| Establishment of an Ebola Treatment Unit and Laboratory —Bombali District, Sierra Leone, July 2014–January 2015 ^63^ | B Gleason et al. 2015, MMWR | 20 |
| Ebola Virus Disease in Children, Sierra Leone, 2014–2015 ^64^ | F Fitzgerald et al. 2016, Emerging Infectious Diseases | 25 |
| Evidence for a Decrease in Transmission of Ebola Virus — Lofa County, Liberia,June 8–November 1, 2014^65^ | A Sharma et al. 2014, MMWR | 18 |
| Evidence for Declining Numbers of Ebola Cases — Montserrado County, Liberia, June–October 2014^66^ | T Nyenswah et al. 2014, MMWR | 20 |
| The natural history of acute Ebola Virus Disease among patients managed in five Ebola treatment units in West Africa: A retrospective cohort study^67^ | K Skrable et al. 2017, PLOS Neglected Tropical Diseases | 26 |
| Minimally Symptomatic Infection in an Ebola `Hotspot': A Cross-Sectional Serosurvey ^68^ | ET Richardson et al. 2016, PLOS Neglected Tropical Diseases | 26 |
| Network visualization for outbreak response: Mapping the Ebola Virus Disease(EVD) chains of transmission in N’Ze´re´kore´,Guinea ^69^ | C Valenica et al. 2016, Elsevier Journal of Infection | 21 |
| Rapid Response to Ebola Outbreaks in Remote Areas — Liberia, July–November 2014^70^ | F Kateh et al. 2015, MMWR | 17 |
| Successful Control of Ebola Virus Disease:Analysis of Service Based Data from Rural Sierra Leone^71^ | K Lokuge et al. 2016, PLOS Neglected Tropical Diseases | 26 |
| The 2014 Ebola virus disease outbreak in Pujehun, Sierra Leone: epidemiology and impact of interventions^72^ | M Ajelli et al. 2015, BMC Medicine | 22 |
| The impact of active surveillance and health education on an Ebola virus disease cluster — Kono District, Sierra Leone, 2014–2015 ^73^ | TS Ariza et al. 2016, BMC Infectious Diseases | 17 |
| Transmission dynamics and control of Ebola virus disease outbreak in Nigeria, July to September 2014^74^ | FO Fasina et al. 2014, Eurosurveillance | 17 |
| Update: Ebola Virus Disease Epidemic — West Africa, January 2015 ^75^ | CDC 2015, MMWR | 13 |
| Notes from The Field: Ebola Virus Disease Cluster - Northern Sierra Leone, January 2016 ^76^ | C Alpren et al. 2016, MMWR | 17 |
| Clinical features of suspected Ebola cases referred to the Moyamba ETC, Sierra Leone: challenges in the later stages of the 2014 outbreak ^77^ | J Arranz et al. 2015, BMC Infectious Diseases | 25 |
| The predictor of mortality outcome in adult patients with Ebola virus disease during the 2014–2015 outbreak in Guinea ^78^ | MS Cherif et al. 2017, European Journal of Clinical Microbiology and Infectious Diseases | 24 |
| Ebola virus disease in children during the 2014–2015 epidemic in Guinea: a nationwide cohort study^79^ | MS Cherif et al. 2017, European Journal of Pediatrics | 26 |
| Cluster of Ebola Virus Disease Linked to a Single Funeral — Moyamba District, Sierra Leone, 2014^80^ | KG Curran et al. 2016, MMWR | 19 |
| Ebola outbreak in rural West Africa: epidemiology, clinical features and outcomes ^81^ | S Dallatomasina et al. 2015, Tropical Medicine and International Health | 20 |
| Epidemiology and Risk Factors for Ebola Virus  Disease in Sierra Leone—23 May 2014 to  31 January 2015^82^ | PM Dietz et al. 2015, Clinical Infectious Diseases | 21 |
| Clinical Features and Outcome of Ebola Virus Disease in Pediatric Patients: A Retrospective Case Series^83^ | M Damkjær et al. 2016, Journal of Pediatrics | 21 |
| Transmission dynamics of Ebola virus disease and intervention effectiveness in Sierra Leone^84^ | LQ Fang et al. 2016, Proceedings of the National Academy of Sciences of the United States of America | 24 |
| Chains of transmission and control of Ebola virus disease in Conakry, Guinea, in 2014: an observational study^85^ | Faye et al. 2015, Lancet | 21 |
| Clinical presentations and outcomes of patients with Ebola virus disease in Freetown, Sierra Leone^87^ | YJ Ji et al. 2016, Infectious Diseases of Poverty | 21 |
| Anatomy of a Hotspot: Chain and Seroepidemiology of Ebola Virus Transmission, Sukudu, Sierra Leone, 2015–16^87^ | JD Kelly et al. 2018, Journal of Infectious Diseases | 24 |
| Clinical features of patients isolated for suspected Ebola virus disease at Connaught Hospital, Freetown, Sierra Leone: a retrospective cohort study^88^ | M Lando et al. 2015, Lancet | 25 |
| Clinical Manifestations and Modes of Death among Patients with Ebola Virus Disease, Monrovia, Liberia, 2014^89^ | LM Mobula et al. 2018, The American Journal of Tropical Medicine and Hygiene | 22 |
| Ebola Virus Disease, Democratic Republic of the Congo, 2014^90^ | C Nanclares et al. (2016), Emerging Infectious Diseases | 21 |
| Clinical profile and containment of the Ebola virus disease outbreak in two large West African cities, Nigeria, July–September 2014^91^ | C Ohuabunwo et al. 2016, International Journal of Infectious Diseases | 23 |
| Field investigation with real-time virus genetic characterisation support of a cluster of Ebola virus disease cases in Dubreka, Guinea, April to June 2015^92^ | A Pini et al. 2018, Eurosurveillance | 20 |
| Clinical Features of Patients With Ebola Virus Disease in Sierra Leone^93^ | E Quin et al. 2015, Clinical Infectious Diseases | 20 |
| Twenty-one days of isolation: A prospective observational cohort study of an Ebola-exposed hot zone community in Liberia ^94^ | GS Williams et al. 2015, Journal of Infectious Diseases | 15 |
| Epidemiologic characteristics, clinical manifestations, and risk factors of 139 patients with Ebola virus disease in western Sierra Leone^95^ | Z Xu et al. 2015, The American Journal of Infectious Diseases | 20 |
| Clinical characteristics of 154 patients suspected of having Ebola virus disease in the Ebola holding center of Jui Government Hospital in Sierra Leone during the 2014 Ebola outbreak^96^ | T Yan et al. 2015, European Journal of Clinical Microbiology and Infectious diseases | 19 |
| Rapid Intervention to Reduce Ebola Transmission in a Remote Village — Gbarpolu County, Liberia, 2014^97^ | DJ Blakely et al. 2015, MMWR | 14 |
| Update: Ebola virus disease epidemic--West Africa, November 2014^98^ | CDC 2014, MMWR | 12 |
| Update: Ebola virus disease epidemic - West Africa, February 2015^99^ | CDC, 2015, MMWR | 11 |
| Elimination of Ebola Virus Transmission in Liberia - September 3, 2015^100^ | L Bawo et al. 2015, MMWR | 11 |
| The Contribution of Ebola Viral Load at Admission and Other Patient Characteristics to Mortality in a Médecins Sans Frontières Ebola Case Management Centre, Kailahun, Sierra Leone, June–October 2014^101^ | F Fitzpatrick et al. 2015, Journal of Infectious Diseases | 22 |
| Community quarantine to interrupt Ebola virus transmission - Mawah Village, Bong County, Liberia, August-October, 2014^103^ | T Nyenswah et al. 2015, MMWR | 15 |
