# Supplementary figures and images for: Reporting quality of the 2014 Ebola outbreak in Africa: A systematic analysis
Source: PLoS One. 2019 Jun 25;14(6):e0218170. doi: 10.1371/journal.pone.0218170 (PMC6592536; doi:10.1371/journal.pone.0218170)

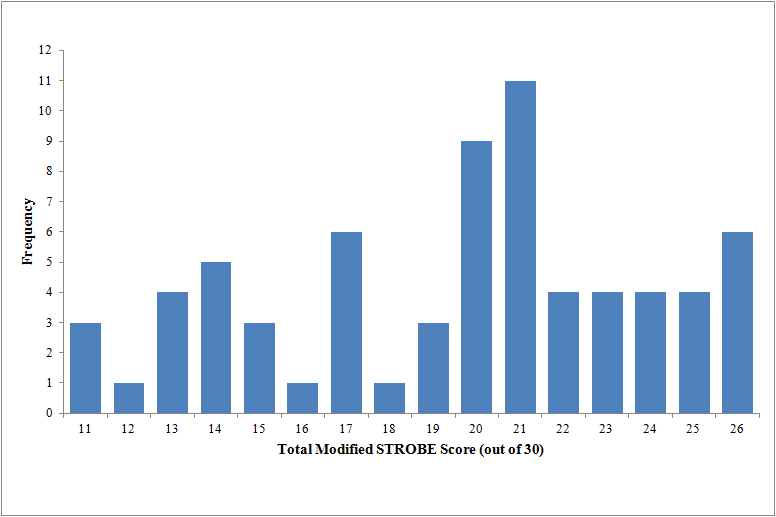

Supplement: S1 Fig — (TIF) [file pone.0218170.s003.tif]
